# Biochemical characterization and cytotoxic effect of the skin secretion from the red-spotted Argentina frog *Argenteohyla siemersi* (Anura: Hylidae)

**DOI:** 10.1590/1678-9199-JVATITD-2019-0078

**Published:** 2020-03-30

**Authors:** Luciano S. Fusco, Rodrigo Cajade, Jose M. Piñeiro, Ana M. Torres, Igor R. F. da Silva, Stephen Hyslop, Laura C. Leiva, Daniel C. Pimenta, Soledad Bustillo

**Affiliations:** 1 Protein Research Laboratory (LabInPro), IQUIBA-NEA CONICET, National University of the Northeast, Corrientes, Argentina.; 2 Herpetology Laboratory, National University of the Northeast, Corrientes, Argentina.; 3 Natural Products Laboratory, National University of the Northeast, Corrientes, Argentina.; 4 Department of Pharmacology, School of Medical Sciences, State University of Campinas (UNICAMP), Campinas, SP, Brazil.; 5 Laboratory of Biochemistry and Biophysics, Butantan Institute, São Paulo, SP, Brazil.

**Keywords:** Argenteohyla siemersi, Hylidae, Skin secretion, Tree frog, Cytotoxicity

## Abstract

**Background:**

*Argenteohyla siemersi* (red-spotted Argentina frog) is a casque-headed tree frog species belonging to the Hylidae family. This species has a complex combination of anti-predator defense mechanisms that include a highly lethal skin secretion. However, biochemical composition and biological effects of this secretion have not yet been studied.

**Methods:**

The *A. siemersi* skin secretion samples were analyzed by mass spectrometry and chromatographic analysis (MALDI-TOF/MS, RP-HPLC and GC-MS). Proteins were also studied by SDS-PAGE. Among the biological activities evaluated, several enzymatic activities (hemolytic, phospholipase A_2_, clotting, proteolytic and amidolytic) were assessed. Furthermore, the cytotoxic activity (cytolysis and fluorescence staining) was evaluated on myoblasts of the C2C12 cell line.

**Results:**

The MALDI-TOF/MS analysis identified polypeptides and proteins in the aqueous solution of *A. siemersi* skin secretion. SDS-PAGE revealed the presence of proteins with molecular masses from 15 to 55 kDa. Steroids, but no alkaloids or peptides (less than 5 KDa), were detected using mass spectrometry. Skin secretion revealed the presence of lipids in methanolic extract, as analyzed by CG-MS. This secretion showed hemolytic and phospholipase A_2_ activities, but was devoid of amidolytic, proteolytic or clotting activities. Moreover, dose-dependent cytotoxicity in cultured C2C12 myoblasts of the skin secretion was demonstrated. Morphological analysis, quantification of lactate dehydrogenase release and fluorescence staining indicated that the cell death triggered by this secretion involved necrosis.

**Conclusions:**

Results presented herein evidence the biochemical composition and biological effects of *A. siemersi* skin secretion and contribute to the knowledge on the defense mechanisms of casque-headed frogs.

## Background

Anuran skin secretions represent a rich source of biologically active compounds such as biogenic amines, alkaloids, bufadienolides, steroids, lipids, peptides and proteins [1-3]. These molecules secreted by the skin glands perform diverse functions including defense against predators [4, 5], prevention of pathogens microbial proliferation [6, 7], reduction of evaporative water loss [8-10], and presumably a critical role in anuran communication [11, 12]**.**

Several studies have investigated the composition [2, 3, 13] and biological activities of skin secretions of anuran amphibians [14, 15]. Remarkable among them is a recent study focused on the phylogenetic analysis and taxonomic revision of “casque-headed frogs” [16]. These hylid frogs belong to the Lophyohylini tribe and includes 85 species from the genera *Aparasphenodon*, *Trachycephallus*, *Corythomantis*, *Dryaderces*, *Itapotihyla*, *Nyctimantis*, *Osteocephallus*, *Osteopilus*, *Phyllodytes, Phytotriades*, *Tepuihyla* and *Argenteohyla* [17]. Characterized by a great morphological diversity (e.g. different degrees of cranial skin co-ossification, colorations and sizes), life histories (e. g. diverse reproductive modes and behaviors) and broad geographic distribution, these neotropical frogs caught the attention of scientists on account of presenting a biologically remarkable skin interaction with the environment. 

Frog skin research focuses on two main themes: 1) the skin as a device to reduce water loss by evaporation and 2) the skin as a defense stratagem. The consolidated group, within the Lophyohylinae subfamily, of the genera *Aparasphenodon, Argenteohyla, Corythomantis* and *Nyctimantis*, has been the most studied as to their skin characteristics [16, 18]. In this sense, cutaneous secretions of the species *Aparasphenodon brunoi* and *Corythomantis greningii* have been implicated in reducing water loss [19, 20] and also as a defensive mechanism [21, 22] as was *Argenteohyla siemersi* [23].

*A. siemersi*, the southernmost species of casque-headed frogs, is found in Argentina, Paraguay and Uruguay [17]. As described for *C. grenningi* and *A. brunoi* [21, 22], *A. siemersi* is considered a truly venomous frog on account of possessing a specific delivery device for its highly lethal skin secretion. This device is formed by dermal bone spines on the surface of the cranial skin associated with the venom glands and is part of a complex combination of several anti-predator mechanisms [23]. Moreover, the high toxicity of this skin secretion is signalized by prominent aposematic coral-reddish spots on several parts of the body [23]. 

Recent insights about the defensive mechanisms in casque-headed frogs provided the basis for deepening the characterization of skin secretions. This new approach began with the biochemical characterization and study of the biological effects of *C. greening* skin secretions that induce a rapid and persistent edema accompanied by an intense dose-dependent nociception [5].

Recently, Cajade et al. [23] determined the lethal dose of *A. siemersi* skin secretion (4.75 μg/mouse, BALB/c mice). This secretion was demonstrated to be more lethal in comparison with *C. greening* (69.75 μg/mouse; Swiss white mice), but less lethal than *A. brunoi* (3.12 μg/mouse, Swiss white mice) [22]. 

Motivated by this high toxicity and a desire to elucidate the general composition and toxic effects of this secretion, in this work we characterized the skin secretion of *A. siemersi* biochemically and described its cytotoxicity in C2C12 myoblast cells. These studies are significant for elucidating the defense mechanisms of casque-headed frogs in an evolutionary context.

## Materials and Methods

### Reagents and venom

Acridine orange, agarose, azocasein, benzoyl-D,L-arginine-*p*-nitroanilide (BApNA), ethidium bromide, fetal bovine serum, hexadecyltrimethylammonium bromide, hyaluronic acid (human umbilical cord) and 4-nitro-3-octanoyloxy-benzoic acid were purchased from Sigma-Aldrich Chemical Co. (St. Louis, MO, USA). Tris base and reagents for electrophoresis were from GE Life Sciences (Piscataway, NJ, USA). Dulbecco’s minimum essential medium (DMEM) and other reagents for cell culture were from Gibco (Buenos Aires, Argentina). Salts for buffers and all other reagents were of analytical grade and were obtained from Merck (Darmstadt, Germany).

The venom of the South American rattlesnake (*Crotalus durissus terrificus*) used as positive control in the assay for phospholipase A_2_ (PLA_2_) was obtained from “Parque Aguará” (San Cosme, Corrientes, Argentina, 27°20'26.4"S 58°35'47.9"W).

### Collection of specimens and skin secretion

**Figure 1.**
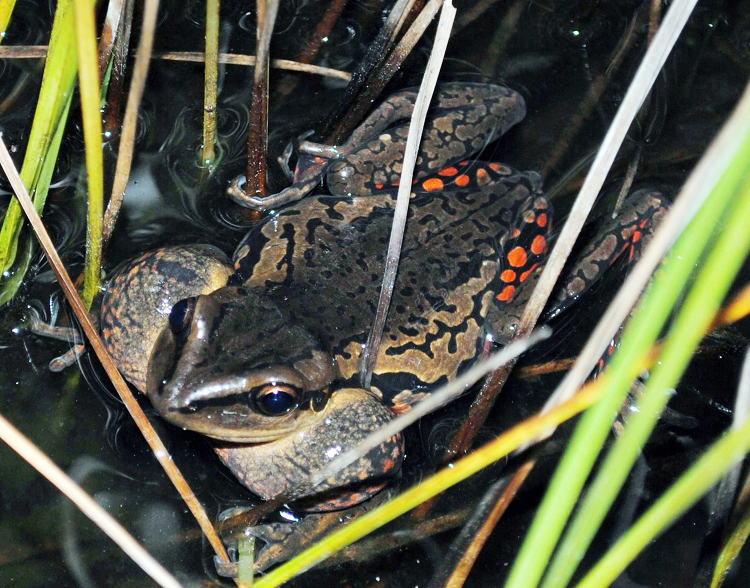
*Argenteohyla siemersi* (male) emitting its advertisement call from the water surface. Note the aposematic coloration (red spots) of the hind legs.

Adult specimens of*A. siemersi* (Fig. 1) were collected in the Rincón Santa María Natural Reserve, Argentina (27°31´31.62″S, 56°36´18.65″W). Cutaneous secretions were obtained as described by Jared et al. [21]. Briefly, skin secretions were obtained by immersing five individuals in ultrapure water and manually stimulating the skin by rubbing the entire body for 5 min. The resulting milky solution was lyophilized, pooled and stored at -70°C. The extraction procedure was approved by the Ethics Committee of the Northeast National University (Res. 0968/18 C.D).

### Mass spectrometry and chromatographic analysis

*Sample processing*

One milligram of *A. siemersi* skin secretion was resuspended in 50 µL of 0.1% trifluoroacetic acid (TFA), vortexed (30 s) and centrifuged (1500 *g*, 3 min). The supernatant was considered the “aqueous solution”. Fifty microliters of methanol was added to the precipitate, vortexed and centrifuged (as previously described) and considered a “methanolic extract”. 

*Mass spectrometry*

Samples were analyzed by matrix-assisted laser desorption ionization time-of-flight mass spectrometry (MALDI-TOF/MS) in an Axima instrument (Shimadzu, Kyoto, Japan), both in linear and reflectron modes (positive ionization mode), using saturated solutions of sinapinic acid and α-cyano as matrices, respectively. The aqueous solution was also processed using C18 ZipTip tips, according to the manufacturer´s instructions. Briefly, a 1 µL sample was pre-mixed with 1 µL of matrix and the mixture applied on the sample holder. Data acquisition and processing were performed with the MALDI-MS Launchpad 2.9.3.20110624 software suite (Shimadzu). Up to 1000 profiles were accumulated, with automatic sample screening. Laser power was set to 120 (arbitrary units). External calibration was done using horse heart myoglobin and porcine insulin (ProteoMass kit, Sigma) as standards.

*Reversed-phase high performance liquid chromatography (RP-HPLC)*

The aqueous solution was analyzed by analytical RP-HPLC. A 5 µL sample was applied to a Discovery C8 column (100 x 2.1 mm, 5 µM) coupled to a binary HPLC system (Shimadzu Proeminence) and the column was eluted at a constant flow of 0.2 mL.min^-1^ with a linear gradient of 0-70% solvent B (solvent A: trifluoroacetic acid:water; 1:1000; solvent B: trifluoroacetic acid:acetonitrile; 1:1000) for 22 min, after isocratic elution for 5 min. UV detection was performed at 214nm (Shimadzu lamp).

*Gas chromatography-mass spectrometry (GC-MS)*

The methanolic extract was analyzed by gas chromatography, coupled to mass spectrometry (GC-MS; model7890A/5975C, Agilent Technologies). A 5 µL sample was injected in the splitless mode with an injector at a temperature of 120°C. Molecules were separated on an HP-5MS column (30 m x 0.25 mm, 0.25 µm - Agilent Technologies), with the oven temperature programmed from 100°C (isothermal for 2 min) to 300°C in 20 min. Helium was used as the carrier gas at a flow rate of 1.5 mL/min. Mass spectra were obtained through electron impact (70 eV) and, after manual checking, the results were compared through the National Institute of Standards and Technology database (http://www.nist.gov/pml/data/asd.cfm) to determine the molecules’ identity.

### SDS-PAGE

The electrophoretic profile of the secretion was assessed by sodium dodecyl sulfate polyacrylamide gel electrophoresis (SDS-PAGE) on 12% polyacrylamide slab gels [24]. The fractions were heated to 100 °C in reducing (β-mercaptoetanol 1%) and non-reducing conditions for 5 min prior to application to the gels that were then run at 40 mA for 1 h. Bromophenol blue was used as a tracking dye. At the end of the run, the gels were subjected to silver staining. 

### Enzymatic activities

All enzymatic assays were performed using the pooled lyophilized milky solution (section “Collection of specimens and skin secretion”). The dry sample was resuspended in phosphate-buffered saline (PBS) solution for analysis. Assays were run in triplicate.

*Hemolytic activity*

Hemolytic activity was assessed as described by Gutiérrez et al. [25]. Briefly, 0.3 mL of packed sheep erythrocytes washed four times with saline solution, 0.3 mL of egg yolk diluted 1:4 with saline solution and 0.25 mL of 0.01 M CaCl_2_ solution were added to 25 mL of 0.8% agarose dissolved in phosphate-buffered saline solution, pH 8.1. The mixture was applied to plastic plates (135 x 80 mm) and allowed to gel after which 3 mm diameter wells were filled with 15 µL of skin secretion solution (0.625-10 mg/mL). The plates were incubated at 37°C for 20 h and the diameters of the hemolytic halos were measured. As negative control, 15 µL of PBS solution was tested. 

*PLA*_*2*_ *activity*

PLA_2_ activity was assayed in 96-well plates, as described by Ponce Soto et al. (2002) [26]. The standard assay mixture contained 200 µL of buffer (10 mMTris-HCl, 10 mM CaCl_2_ and 100 mM NaCl, pH 8), 20 µL of substrate (3 mM 4-nitro-3-octanoyloxy- benzoic acid) and 20 µL of buffer or skin secretion (1.6-100 µg) in a final reaction volume of 240 µL. The mixture was incubated for 30 min at 37 ºC, and the absorbance at 425 nm was recorded at 10-min intervals. Enzymatic activity, expressed as the initial reaction velocity (V_0_, nmoles/min), was calculated based on the increase in absorbance after 20 min. PBS and *C.d.terrificus* venom were used as negative and positive controls, respectively.

*In vitro clotting activity*

The clotting activity of the secretion was evaluated using a Wiener Lab Fibrintimer 2® coagulometer (Germany). Briefly, 75 µL of secretion (1mg/mL in NaCl 0.8%) was added to 75 µL of sheep plasma and the time for clot formation was measured. PBS and thrombin from human plasma (Merck®) were used as negative and positive controls, respectively. 

*Proteolytic activity*

Skin secretion was assayed for proteolytic activity using azocasein as substrate [27]. Secretion aliquots of 25 μL (125 μg) were added to 142 μL of azocasein (5 mg/mL) in 50 mM Tris-HCl, pH 8, followed by incubation at 37°C for 90 min. Undigested azocasein was precipitated by adding 334 μL of 10% trichloroacetic acid (TCA) to the reaction mixture followed by centrifugation (1300 *g*, 5 min, room temperature). The supernatants (100 μL) were transferred to a 96-well plate containing an equal volume of 0.5 M NaOH and the resulting absorbances were read in a microplate reader (Thermo Scientific) at 450 nm. An increase in absorbance indicated the presence of proteolytic activity. The blank was prepared by precipitating the substrate plus the sample in TCA without prior incubation of the sample-substrate mixture.

*Amidolytic activity*

The chromogenic substrate benzoyl-D,L-arginine-*p*-nitroanilide (BApNA) was employed to determine the amidolytic activity of the secretion [28]. This activity was measured by incubating 20*μL* (100*μ*g) of skin secretion with 200*μ*L of solution containing 1% BApNA in 100 mM Tris-HCl, pH 8.0, at 37°C for 5 h. The increase in absorbance was monitored at 405 nm and the amount of reaction product formed was quantified using a molar extinction coefficient of 8800 M^−1^·cm^−1^ for *p*-nitroanilide. One unit of enzymatic activity was defined as the amount of enzyme able to release 1 μmol of *p*-nitroanilide/min under the described conditions. The negative control consisted of water instead of skin secretion sample.

### Cytotoxicity and morphological analysis

*Cytotoxicity assay*

Undifferentiated myoblasts from C2C12 cells (CRL-1772; American Tissue and Cell Culture - ATCC) were utilized to examine the cytotoxicity of the secretion. The cells were seeded in 96-well plates at an initial density of~1-2 x 10^4^ cells/well, in Dulbecco’s minimum essential medium (DMEM) supplemented with 5% fetal bovine serum (FBS). When the monolayers reached 80-90% confluence, variable amounts of skin secretion were diluted in DMEM-5%FBS (8-2000 µg/mL) and added to the cells in a total volume of 200 µL/well. Cell viability was quantified by crystal violet staining after incubating the cells at 37 ºC in a 5% CO_2_ atmosphere for 3 h [29]. The absorbance of the released dye was read at 620 nm and the percentage of cell viability was calculated. The secretion concentration that killed 50% of cells was defined as the cytotoxic concentration 50% [29].

Cytolysis was assessed by monitoring the release of the cytosolic marker enzyme lactate dehydrogenase (LDH) using a commercial kit (Wiener®, LDH-P UV, Buenos Aires, Argentina), as previously described [30]. Reference controls for 0% and 100% cytolysis consisted of medium alone and medium from cells incubated with 0.1% Triton X-100 (v/v), respectively. All assays were done in triplicate. Morphological alterations and cell damage were assessed qualitatively by light/phase contrast microscopy (Axiovert 40^®^, Carl Zeiss, Argentina) and investigated using a light phase contrast microscope (Axiovert40^®^), in which photographs were taken with a digital camera (Canon CCD 2272x1704).

*Fluorescence staining*

The mechanism of cell death triggered by the skin secretion was assessed by dual staining with acridine orange/ethidium bromide (AO/EB). For this, myoblast cells were grown on cover slips and incubated with 150 or 300 μg of secretion/mL for 3h in a 5% CO_2_ atmosphere at 37°C. These concentrations were selected based on the previously described cytotoxicity assay. PBS was used in the negative control assays. After incubation, myoblasts were washed twice with PBS and then gently mixed with a solution of AO and EB (1 μg/mL each) for 1 min [31]. Coverslips were then applied to the slides and the sections were examined and photographed with a fluorescence microscope (Axioskop 40®/Axioskop 40 FL®, Carl Zeiss). The standard blue filter of the microscope was used, which includes a wide blue band excitation filter of 450-480 nm, a dichromatic mirror of 500 nm and a barrier filter of 515 nm.

### Statistical analysis

Quantitative data are shown as the mean values ± standard deviation (SD) of at least three independent experiments. Statistical comparisons were made using one-way ANOVA followed by the Tukey (Honestly Significant Difference) test, with p < 0.05 indicating significance. All test assumptions were verified.

## Results

### Mass spectrometry and chromatographic analysis

*Mass spectrometry*

**Figure 2.**
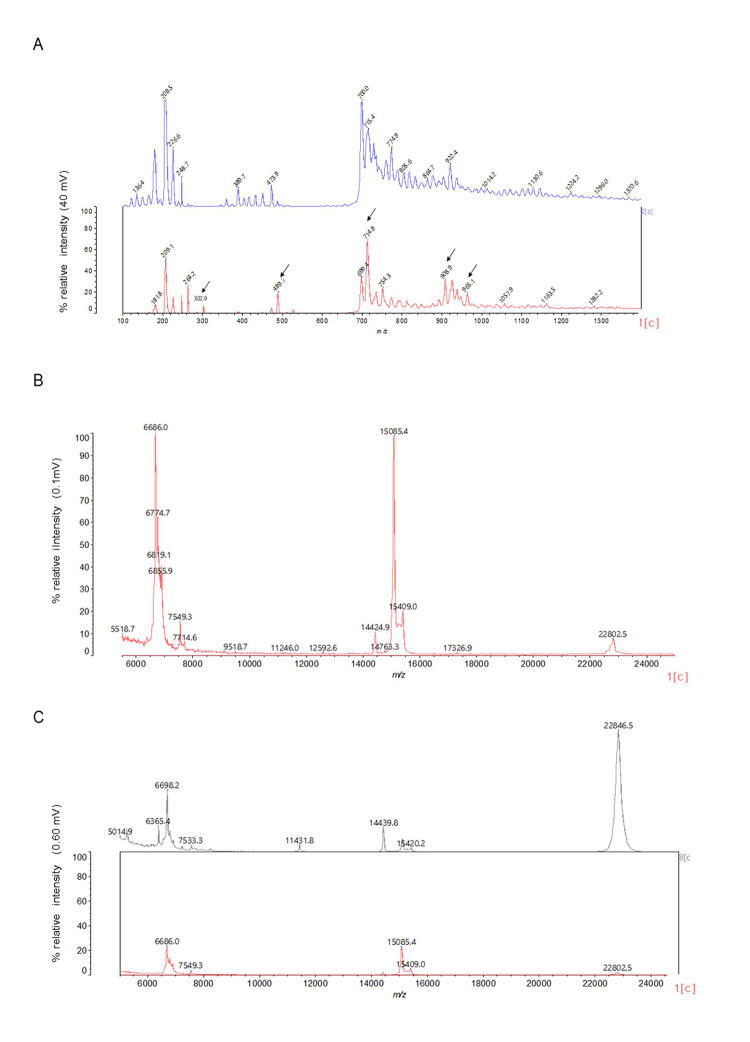
(A) Low-molecular-mass MALDI-TOF profile of the aqueous skin secretion solution of *A. siemersi*. Lower profile (red): actual sample; upper profile (blue): sinapinic acid (matrix). The sample exclusive peaks are indicated (arrows). (B) High-molecular-mass MALDI-TOF profile of the C18 ZipTip processed aqueous skin secretion solution of *A. siemersi*, indicating the presence of large peptides and proteins. (C) High-molecular-mass MALDI-TOF profile of the aqueous skin secretion solution of *A. siemersi*. Lower profile (red): C18 ZipTip processed sample; upper profile (grey): unprocessed aqueous skin secretion solution.

MALDI-TOF/MS analysis of aqueous solution revealed the presence of few low-molecular-mass components (Fig. 2A). The observed *m/z* values ranged from ~300 to ~900 Da. On the other hand, the high-molecular-mass profile of the secretion (Fig. 2B) showed the presence of three major molecular groups: a) peptides in the 6-7 kDa range, b) low-molecular-mass proteins in the 14.4-15.5kDa range and c) a 22 kDa protein. This profile did not change qualitatively after C-18 ZipTip sample enrichment [Fig. 2C, Lower profile (red): C18 ZipTip processed sample; upper profile (grey): unprocessed aqueous solution]. Indeed, C18-ZipTip processing led to loss of a ~5 kDa peptide and a significant reduction in the signalization of the 22 kDa protein. C4-ZipTip sample processing was also done but had a markedly adverse effect on the secretion profile (data not shown).

*RP-HPLC*

**Figure 3.**
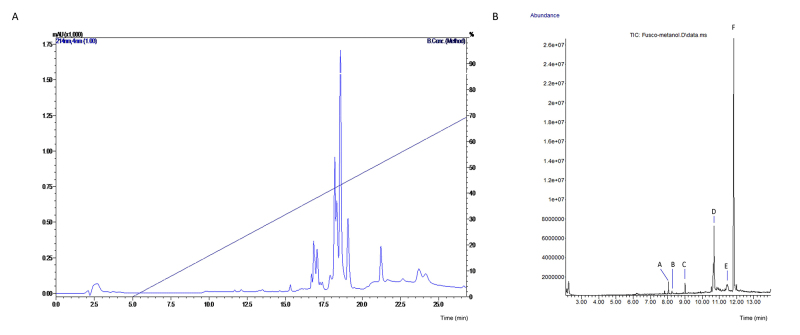
(A) C8-RP-HPLC profile of the aqueous skin secretion solution of *A. siemersi*: UV detection was performed at 214 nm. (B) Total ion chromatography (TIC), profile of the GC separation of the *A. siemersi* skin secretion methanol extract. The main peaks obtained were identified as fatty acid (A, C and D) or bufadienolide steroids (B, E and F).

The aqueous solution was chromatographed by RP-HPLC on a C8 column, as shown in Fig. 3A. The complexity of the profile is in concordance with the MALDI-TOF/MS profile, with major peaks at retention times of 17, 19 and 22 min. 

*Gas chromatography-mass spectrometry (GC-MS)*

The GC-MS profile of the methanolic extract (Fig. 3B, total ion chromatography, TIC) revealed a relatively simple composition. The fragmentation spectra (Additional file 1) showed a predominance of lipids, particularly fatty acids and bufadienolide-like steroids.

### SDS-PAGE

The electrophoretic protein profile of *A. siemersi* skin secretion exhibited numerous bands with molecular masses from 15 to 55 kDa. The estimated masses were 15.24, 22, 27.51, 34, 45, 55 kDa and the profile was practically not affected by the reducing agent (Additional file 2).

### Enzymatic activities

*Hemolytic activity*

**Figure 4.**
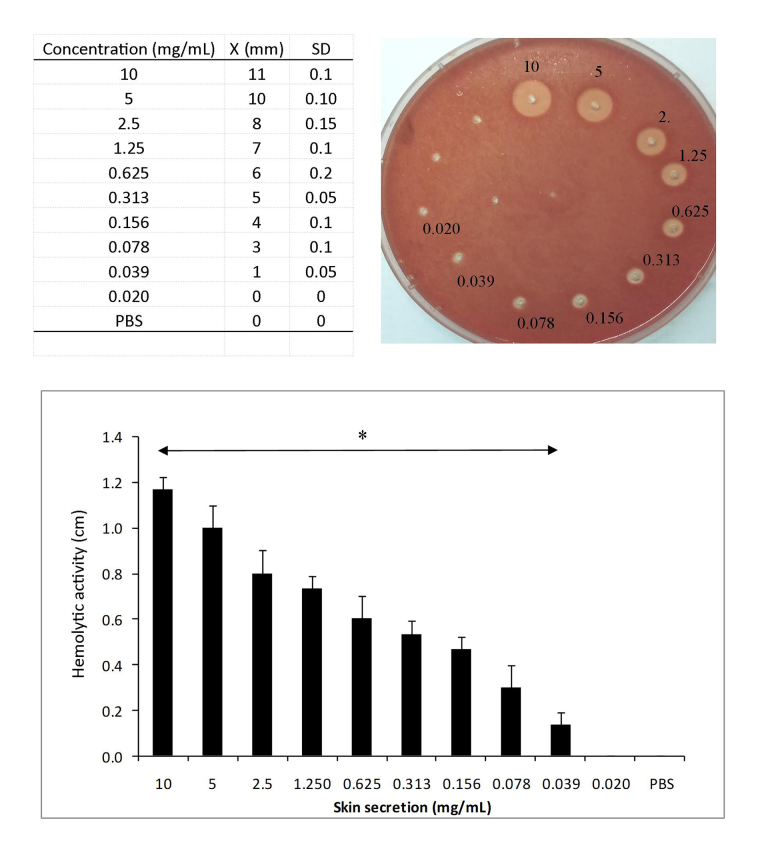
Hemolytic activity of *A. siemersi* skin secretion. Aliquots (15 µL) of secretion (0.02-10 mg/mL) were added to wells cut in agarose-erythrocyte plates and incubated for 20h at 37°C, after which the diameters of the hemolytic halos were measured. The columns represent the mean ±SD of three independent experiments (*p < 0.05), compared to the diameters of control halos (wells that received only PBS).

The *A. siemersi* skin secretion showed hemolytic activity at all concentrations tested (Fig. 4). The formation of halos in agarose-erythrocyte gels was directly proportional to the secretion concentrations assayed and significantly different from the control (*p < 0.05). Maximum activity was evidenced by a 11 ± 0.1 mm halo (10 mg/mL) and the lowest recorded halo measured 4.1 ± 0.05 mm (0.039 mg/mL).

*PLA*_*2*_ *activity*

**Figure 5.**
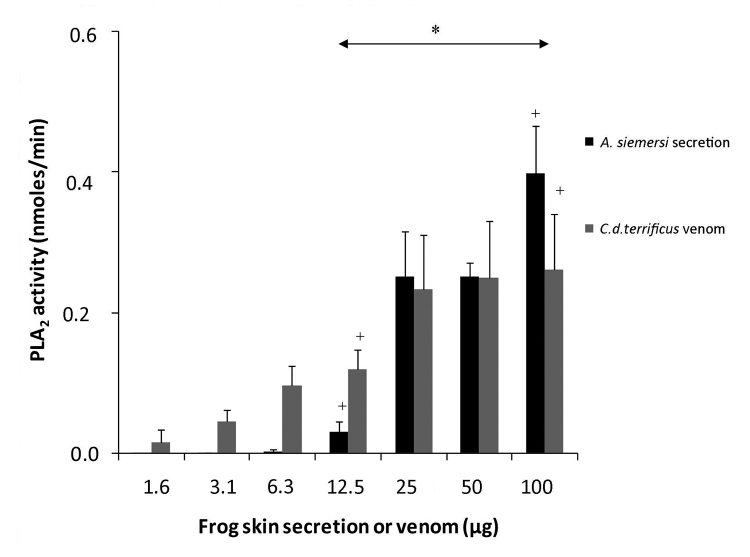
PLA_2_ activity of *Argenteohyla siemersi* skin secretions (1.6-100 µg) was measured using 4-nitro-3-octanoyloxy- benzoic acid substrate in 10 mM Tris-HCl, 10 mM CaCl2, 100 mM NaCl buffer (pH 8) in a final reaction volume of 240 µL. Columns represent the mean ± SD of three independent experiments, ∗p < 0.05 PLA_2_ activities of *A. siemersi* skin secretions vs. control (PBS) and + p < 0.05 PLA_2_ activities of *Argenteohyla siemersi* skin secretions vs. *C. d. terrificus* venom.

PLA_2_ activity was verified in *A. siemersi* skin secretion. The lowest concentrations tested (1.6 - 6.3 μg) showed no enzymatic effect, but quantities equal to or higher than 12.5 μg evidenced PLA_2_ activity (*p ?λτ; 0.05 versus PBS control) and were compared to those exhibited by *C. d. terrificus* venom (Fig. 5).

*Clotting, proteolytic and amidolytic activities*

All these activities were negative and demonstrated that *A. simersi* skin secretion did not produce any clotting, proteolytic or amidolytic effect at the concentrations assayed.

### Cytotoxicity and morphological analyses

*Cytotoxicity and morphological analyses*

**Figure 6.**
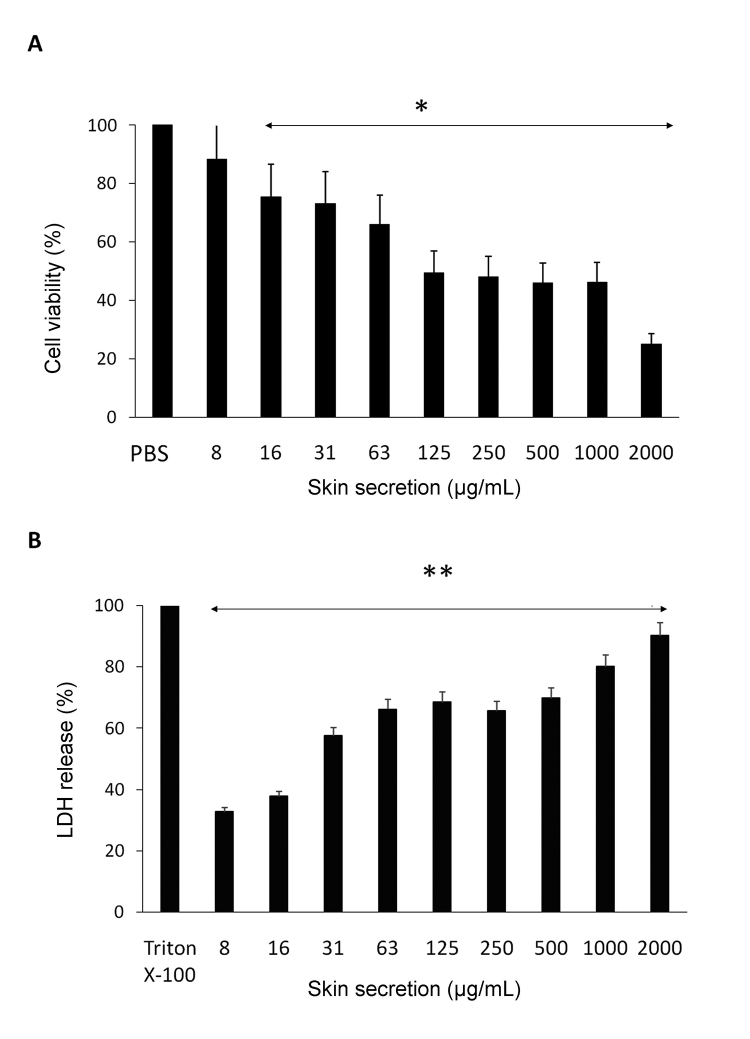
Cytotoxicity of *A. siemersi* skin secretion (8-2000 μg/mL) in C2C12 myoblasts after 3h of incubation. **(A)** Cell viability evaluated by crystal violet staining. **(B)** Release of lactate dehydrogenase (LDH) as an indication of cell membrane damage. The columns in A and B represent the mean ± SD of three independent experiments (*p < 0.05) compared to PBS-treated (control) cells (panel A) and **p < 0.05 compared to the positive control (100% lysis by Triton X-100) (panel B).

**Figure 7.**
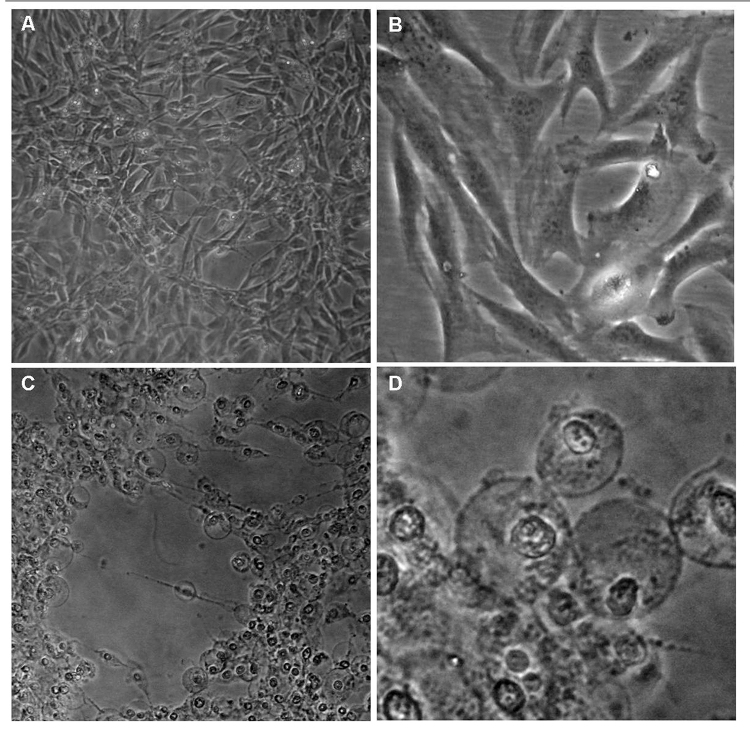
Morphological characterization of murine C2C12 cells under phase contrast microscopy after 3h of incubation with skin secretion of *A. siemersi*. **(A)** Control (x200). **(B)** Control (x400). **(C)** Skin secretion of *A. siemersi* (250 μg/mL - x200). **(D)** Skin secretion of *A. siemersi* (250 μg/mL - x400).

Skin secretion from *A. siemersi* evidenced cytotoxicity against C2C12 cell line in a dose-dependent manner. Staining with crystal violet revealed that the secretion caused the progressive detachment of myoblasts from their substrate at all concentrations tested (Fig. 6A). The cytotoxic concentration 50 (CC_50_) was graphically obtained by linear regression analysis (CC_50_: 257.56 μg/mL, *r* = 0.963). In addition, the release of cytoplasmic lactic dehydrogenase (LDH) indicated disruption of cell membranes after the 3-h incubation (Fig. 6B). Morphological analyses were made by phase-contrast microscopy. Untreated C2C12 cells were homogeneously distributed on the cultured field and exhibited a thin elongated shape (Fig. 7A and B). In contrast, myoblasts exposed to skin secretion evidenced cell alterations that include increase of cellular size, detachment that resulted in extended areas devoid of cells and disruption of several plasma membranes (Fig. 7C and D). All these changes are compatible with necrosis and were more evident at higher doses.

***Fluorescence staining***

**Figure 8.**
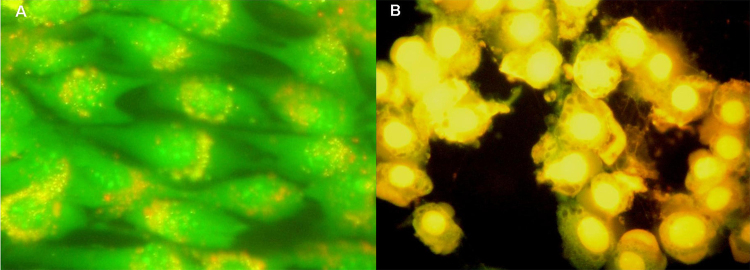
Acridine orange and ethidium bromide fluorescence staining. Myoblasts were grown on coverslides and treated with *A. siemersi* skin secretion for 3h. **(A)** Control (x400). **(B)** Skin secretion of *A. siemersi* (150 μg/mL - x400), nuclei were stained with orange fluorescent dye corresponding to ethidium bromide, indicating compromised membrane integrity.

In order to corroborate the cell death mechanism triggered by *A. siemersi* skin secretion, treated myoblast cells were stained with two nucleic acid-binding fluorochromes, namely acridine orange and ethidium bromide. Control untreated cells exhibited a green fluorescence, due to exclusion of ethidium bromide but not of acridine orange. Viable cells showed a light green nucleus with intact structure and presented punctuated orange red fluorescence in the cytoplasm, representing lysosomes stained by acridine orange (Fig. 8A). After 3h of incubation with *A. siemersi* skin secretion, typical features of necrosis were observed at both concentrations assayed (300 and 150 μg/mL). Necrotic cells exhibited orange fluorescent nuclei stained with ethidium bromide, indicating compromised membrane integrity (Fig. 8B). 

## Discussion

Casque-headed frogs represent one of the most recent challenges for scientists due to the study of their complex defensive mechanisms and the biochemical exploration of their skin secretions. A pioneering study on *C. greening* [21] promotes a new field of research in these frogs with much knowledge that remains to be elucidated.

The anti-predator mechanisms of *A. siemersi* were recently described by Cajade et al. [23]. This complex technique consists of behavioral and ecological traits, including secretive and semi-phragmotic habits and postures. Morphological features include cryptic and aposematic colorations, a skull covered with bony dermal spines and protuberances that are associated with two types of granular venom glands.

Although these authors previously demonstrated a highly lethal dose for the skin secretion of this frog, its biochemical characteristics or biological activities have not yet been investigated. Thus, within the present study we have successfully undertaken a general characterization of *A. siemersi* secretion and some of its toxic activities.

Proteins are common components of casque-headed tree frog skin secretions as previously demonstrated in *C. greeningi*and*A. brunoi* [5, 22]. In the present work, MALDI-TOF/MS analysis identified polypeptides and proteins in an aqueous solution of *A. siemersi* skin secretion ranging between 6 and 22 kDa. Although additional analyses are necessary to support these findings, molecular masses could be interpreted as follows: polypeptides in the 6-7.5 kDa range (Kazal-type inhibitors) [32]; proteins in the 14-15.5 kDa range (PLA_2_) [33, 34] and protein of 22 kDa (an Ankyrin repeat domain 55) [35]. Interestingly, we did not detect masses corresponding to peptides (< 5 kDa), compounds that are usually present in tree-frogs skin secretions [7, 36]. The highest molecular weight proteins observed in the SDS-PAGE (> 30 kDa) could not be detected in the MALDI-MS analyses. However, this is not unusual, since a characteristic of this technique is promoting the ionization of lighter or more volatile ions [37].

The biological role of steroids and alkaloids in amphibian skin secretions have been examined in several studies [38-40]. Recently, Mendes et al. (2016) [5] suggested the presence of these two molecules in the skin secretion of *C. greening* [5]. Similarly, another species of the genus *Osteocephalus* proved to have in its cutaneous secretion bufotenin, a tryptamine alkaloid quite common among anurans [41]. In the present work, bufadienolide steroids were detected in the skin secretion of *A. siemersi*, but the presence of molecules compatible with alkaloids could not be confirmed. Additional spectrometric and spectroscopic analyses are required.

*A. siemersi* skin secretion also revealed the presence of some lipids in the obtained methanolic extract. The lipid profile consisted of octadecanoic acid, methyl ester and hexadecanoic acid, as identified in our analyses. This corroborates previous findings by Centeno et al. [42], who reported the presence of this lipid type in *Bokermanohyla alvarengai* (Anura: Hylidae) skin secretion. Many works associated the presence of lipids in secretions from hylid frogs with the reduction in water loss due to evaporation [10, 43]. However, *A. siemersi* inhabits humid environments, which suggests that water availability and temperature do not fluctuate, thus the role of these lipids remains unclear.

Hemolysis can be induced by numerous proteins and peptides derived from animals, plants or microbes. In this sense, several snake, scorpion and bee venoms have shown hemolytic activity, primarily through the action of the PLA_2_ enzymes [44]. This effect has also been demonstrated in amphibians, mainly due to the presence of peptides [45]. Coincidentally with this, our results found that *A. siemersi* skin secretion produced hemolysis and evidenced PLA_2_ activity.

Similar to a previous work regarding the anuran *R. schneideri* parotoid secretion [46], our results showed that *A. siemersi* skin secretion exerted no pro‐coagulant activity. Likewise, no proteolytic or amidolytic activities were detected in the secretion of Argentine frog skin. In contrast, *C. greening* and *A. brunoi* skin secretions showed proteolytic and fibrinolytic activities [22]. Additionally, and also different from our results, proteolytic activity was evidenced in the skin secretion of another frog of the Hylidae family, *Phyllomedusa hypochondrialis,* and related to the physiopathological (edematic and myotoxic) activities observed [34]. 

Previous reports have demonstrated that amphibian skin secretions [5, 47] or isolated toxins/peptides [48, 49] are cytotoxic and exert antiproliferative effects. Herein we confirmed that *A. siemersi* skin secretion caused a dose-dependent decrease in C2C12 myoblast cell viability. Morphological alterations were compatible with the necrosis cell death mechanism and included enlarged cells, formation of cytoplasmic vacuoles and disruption of plasma membrane. This cell membrane rupture resulted in the release of cytoplasmic lactate dehydrogenase (LDH) and confirmed the cytolysis triggered by this cutaneous secretion. Fluorescence staining supported the mechanism herein postulated. Further studies will be required to identify which molecules present in the *A. siemersi* skin secretion are responsible for this cytotoxic effect. 

Electrophoretic analysis of*A. siemersi* skin secretion revealed protein bands with molecular masses between 14 and 50 kDa. On the other hand, the MS analysis showed the existence of two protein populations of 15 and 22 kDa, probably corresponding to those that migrated further in the SDS-PAGE under reducing and non-reducing conditions. The electrophoretic protein profile was similar to the one published for *C. greeningi*secretion, another hylid species [22]. In this sense, bands of ~15, 20, 30, 40 and 55 kDa were common for both secretions, indicating a high degree of interspecific conservation. In contrast, *A. brunoi* skin secretion presented a similar profile only in the range of 40-55 kDa. [22]. 

In conclusion, herein we described for the first time the biochemical characteristics and biological activities of the skin secretion of the red-spotted Argentina frog, *A. siemersi.* Further studies are required to identify the secretion components and molecular mechanisms involved in these activities.

### Abbreviations

AO: acridine orange; ATCC: American Tissue and Cell Culture; BApNA : benzoyl-D,L-arginine-*p*-nitroanilide; CC_50_: Cytotoxic concentration 50; DMEM: Dulbecco’s minimum essential medium; EB: ethidium bromide; FBS: fetal bovine serum; GC-MS: gas chromatography-mass spectrometry; LDH: lactate dehydrogenase; MALDI-TOF/MS: matrix-assisted laser desorption ionization time-of-flight mass spectrometry; MM: molecular mass markers; NR: non reduced condition; PBS: phosphate-buffered saline; PLA_2_: Phospholipase A_2_; R: reduced conditions; RP-HPLC: reversed-phase high performance liquid chromatography; SDS-PAGE: sodium dodecyl sulfate polyacrylamide gel electrophoresis; SD: standard deviation; TIC: total ion chromatography; TCA: trichloroacetic acid; TFA: trifluoroacetic acid; UV: ultraviolet.

## Supplementary material

The following online material is available for this article:

Additional file 1. Gas chromatography-mass spectrometry. **(A)** Total ion chromatography (TIC), profile of the GC separation of the *A. siemersi* skin secretion methanol extract. **(B)** CG-MS proposed/identified molecules present in the methanol extract of *A. siemersi* skin secretion. **(C)** Molecular identification.

Additional file 2. Protein profiles of skin secretions. SDS-PAGE of skin secretions non-reduced (NR) and reduced conditions (R). MM, molecular mass markers (X10-3 Da; markers: phosphorylase b-94, albumin-67, ovalbumin-45, carbonic anhydrase-30, trypsin inhibitor-20.1, a-lactalbumin-14.4).
