# Supplementary material for: Biochemical characterization and cytotoxic effect of the skin secretion from the red-spotted Argentina frog Argenteohyla siemersi (Anura: Hylidae)
Source: J Venom Anim Toxins Incl Trop Dis. 2020 Mar 30;26:e20190078. doi: 10.1590/1678-9199-JVATITD-2019-0078 (PMC7112748; doi:10.1590/1678-9199-JVATITD-2019-0078)
Supplement: Additional file 1. [file 1678-9199-jvatitd-26-e20190078-s1.pdf]

Supplementary Material to "Biochemical characterization and cytotoxic effect of the skin secretion from the red-spotted Argentina frog *Argenteohyla siemersi* (Anura: Hylidae)"

A.

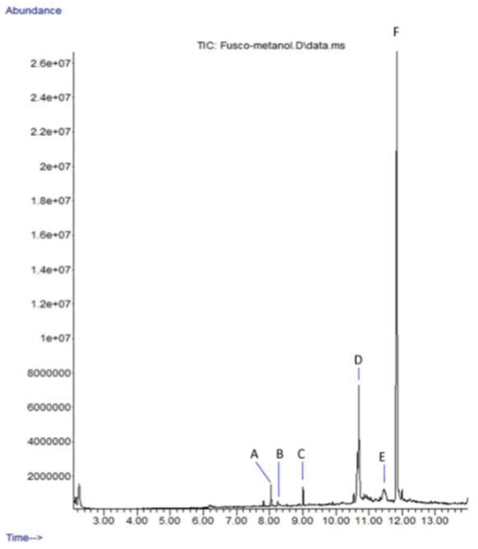

B.

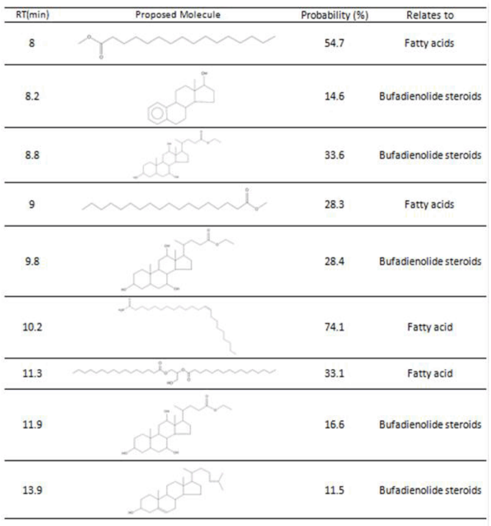

RT(min)	Proposed Molecule	Probability (%)	Relates to
8		54.7	Fatty acids
8.2		14.6	Bufadienolide steroids
8.8		33.6	Bufadienolide steroids
9		28.3	Fatty acids
9.8		28.4	Bufadienolide steroids
10.2		74.1	Fatty acid
11.3		33.1	Fatty acid
11.9		16.6	Bufadienolide steroids
13.9		11.5	Bufadienolide steroids

RT* Retention Time

C.

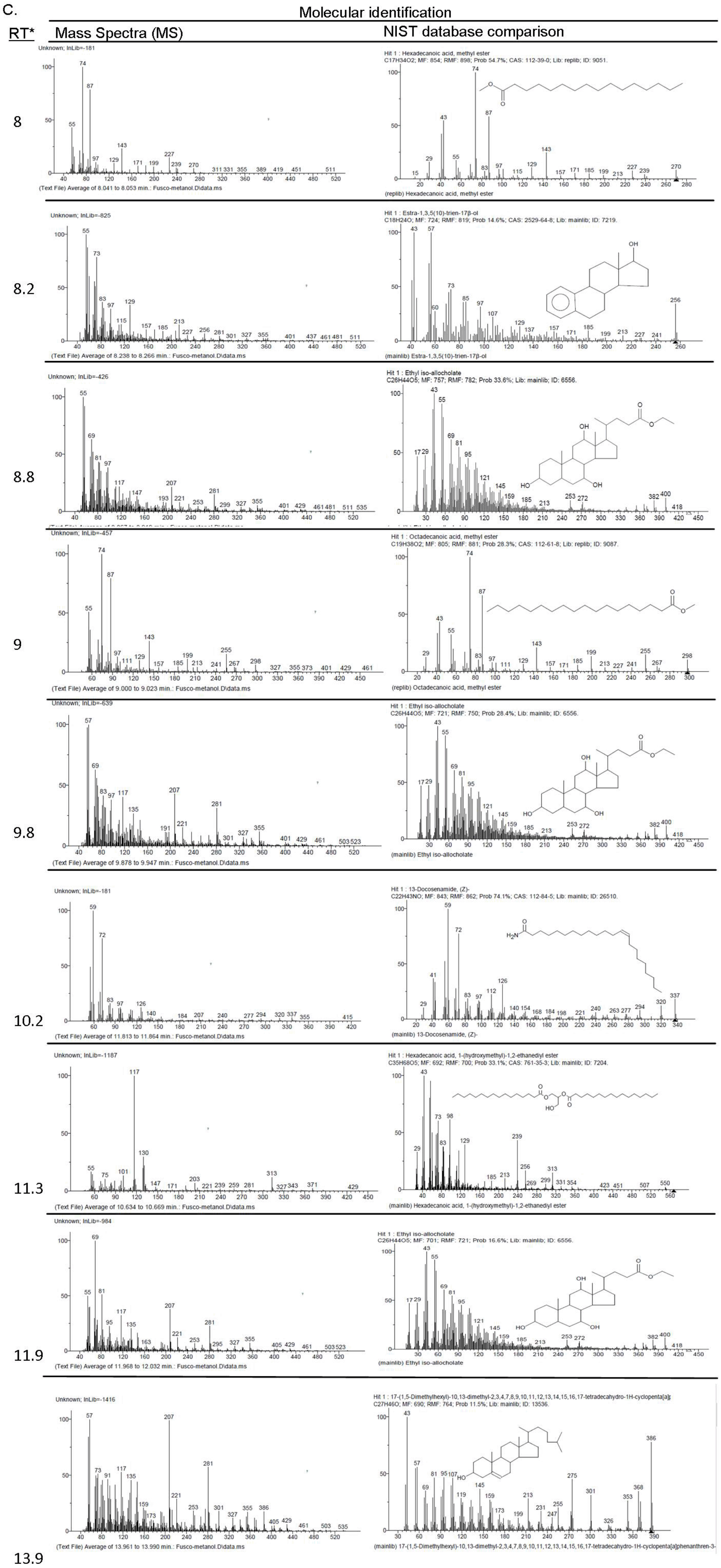

Additional file 1. Gas chromatography-mass spectrometry. **(A)** Total ion chromatography (TIC), profile of the GC separation of the *A. siemersi* skin secretion methanol extract. **(B)** CG-MS proposed/identified molecules present in the methanol extract of *A. siemersi* skin secretion. **(C)** Molecular identification.
