# Supplementary material for: Biochemical characterization and cytotoxic effect of the skin secretion from the red-spotted Argentina frog Argenteohyla siemersi (Anura: Hylidae)
Source: J Venom Anim Toxins Incl Trop Dis. 2020 Mar 30;26:e20190078. doi: 10.1590/1678-9199-JVATITD-2019-0078 (PMC7112748; doi:10.1590/1678-9199-JVATITD-2019-0078)
Supplement: Additional file 2. [file 1678-9199-jvatitd-26-e20190078-s2.pdf]

Supplementary Material to "Biochemical characterization and cytotoxic effect of the skin secretion from the red-spotted Argentina frog *Argenteohyla siemersi* (Anura: Hylidae)"

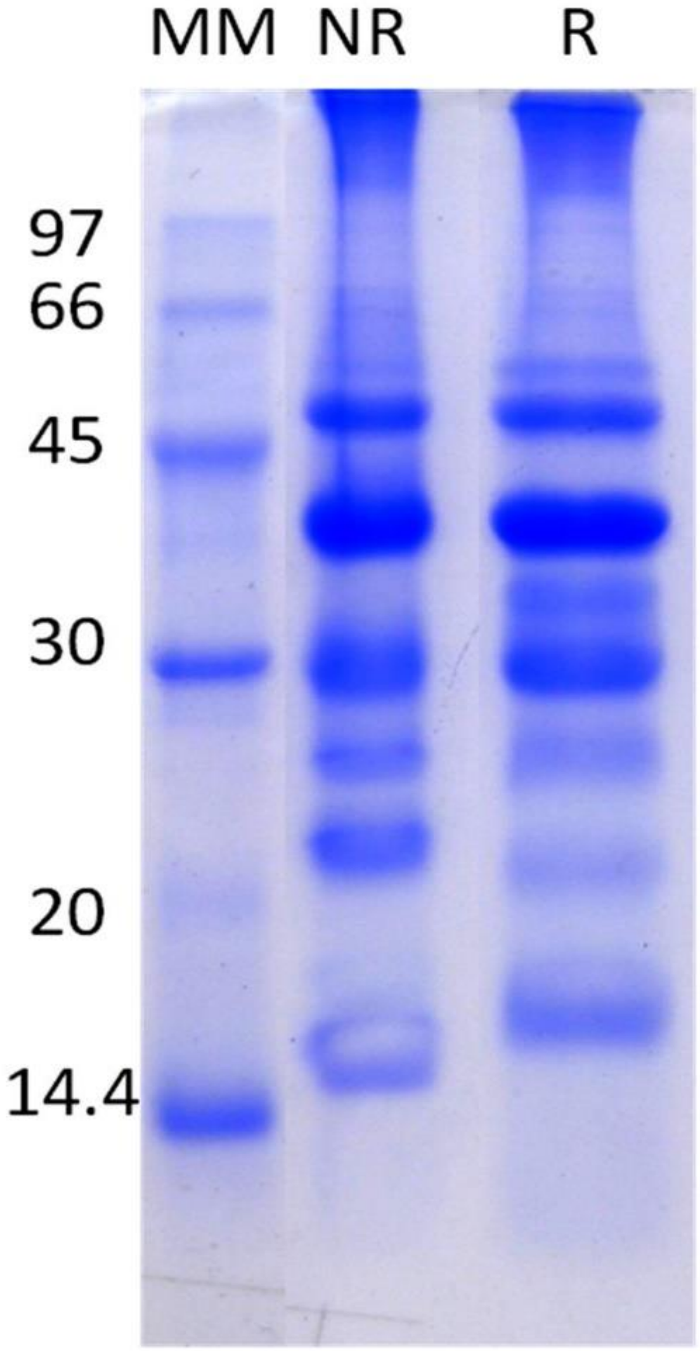

Additional file 2. Protein profiles of skin secretions. SDS-PAGE of skin secretions non-reduced (NR) and reduced conditions (R). MM, molecular mass markers (X10-3 Da; markers: phosphorylase b-94, albumin-67, ovalbumin-45, carbonic anhydrase-30, trypsin inhibitor-20.1, a-lactalbumin-14.4).
